# Association between self‐stigma and self‐compassion in patients with schizophrenia: A longitudinal study from hospital admission to first follow‐up after discharge

**DOI:** 10.1111/jjns.12648

**Published:** 2025-01-15

**Authors:** Keita Toshi, Miharu Nakanishi, Mai Sakai, Hatsumi Yoshii

**Affiliations:** ^1^ Department of Psychiatric Nursing Tohoku University Graduate School of Medicine Sendai‐shi Japan; ^2^ Kanno‐aiseikai Midorigaoka Hospital Shiogama‐shi Japan; ^3^ Department of Psychiatric Nursing Miyagi University Taiwa‐cho, Kurokawagun Japan

**Keywords:** acute psychiatric care ward, internalized stigma

## Abstract

**Aim:**

Self‐stigma is a major factor preventing the recovery of individuals with schizophrenia. Psychosocial interventions can reduce self‐stigma, and mental health nurses may play a crucial role in leading them, but little is known about the modifiable factors that should be targeted. We aimed to investigate the association between self‐stigma and self‐compassion in patients with schizophrenia from admission to the first follow‐up after discharge.

**Methods:**

Twenty‐three patients with schizophrenia were recruited from an acute psychiatric ward in a private psychiatric hospital in Japan. Participants filled out the Japanese versions of the Internalized Stigma of Mental Illness (ISMI) scale, the Self‐Compassion Scale (SCS), and the Positive and Negative Syndrome Scale (PANSS) at the following three time points: 1 month after admission, discharge, and first follow‐up after discharge at outpatient care. We used a linear mixed model to examine the association between self‐stigma, self‐compassion, and the symptoms. In the first model, we used self‐stigma as a dependent variable and included time of assessment and positive and negative symptoms as independent variables. In the second model, we added self‐compassion to the independent variables.

**Results:**

Self‐stigma did not change over time. Regarding the linear mixed model, the first model showed that participants with more positive symptoms tended to report worse self‐stigma (*p* = .052). The second model showed a significant association between increasing self‐stigma and higher over‐identification (*p* = .001).

**Conclusions:**

Our results suggest that interventions focusing on over‐identification can reduce self‐stigma. Nurse‐led intervention programs with a focus on over‐identification should be further developed for effectiveness.

## 1 INTRODUCTION

Schizophrenia is a chronic mental disease that causes long‐term disability. To achieve full recovery, psychosocial treatment should target, beyond clinical recovery, subjective aspects such as personal recovery (Dubreucq et al., 2022). The concept of personal recovery has recently been defined as a life worth living, which is about building a life that is satisfying, fulfilling, and enjoyable (Kasai & Fukuda, 2017). Mental health nurses play a crucial role and are well suited to provide recovery‐oriented services due to their status as the largest group of mental healthcare providers globally (Thongsalab et al., 2023).

One major factor that inhibits the recovery of a person with mental illness is self‐stigma (Oexle et al., 2018; Yanos et al., 2008), which should be identified and addressed in daily observations based on a well‐established nurse–patient relationship. Stigma in mental illness involves societal prejudice, discrimination, and unpleasant experiences among patients with schizophrenia and their families (Rezayat et al., 2019). Self‐stigma (or internalized stigma) occurs when individuals are aware of the negative stereotypes associated with their illness, agree with them, and apply them to themselves (Corrigan et al., 2009). A systematic review and meta‐analysis by Livingston and Boyd (2010) suggest that severe psychiatric symptoms may evoke self‐stigma in people with mental illness. However, a range of psychosocial variables also correlates with self‐stigma (Sarraf et al., 2022). Thus, psychosocial interventions are warranted to reduce self‐stigma in people with mental illness.

Self‐compassion can serve as resilience against self‐stigma in patients with mental illness. People with self‐compassion showed lower levels of self‐stigma (Hilbert et al., 2015; Wong et al., 2016). Self‐compassion is characterized by a self‐caring (self‐kindness), inclusive (common humanity), and compassionate (mindfulness) attitude in the face of hardship. Therefore, self‐compassion may encourage the person to reduce self‐stigmatizing notions including self‐judgment, isolation, and over‐identification. Integrating self‐compassionate approaches into psychosocial interventions might reduce self‐stigma. However, the underlying pathways between self‐stigma and self‐compassion have not been clarified (Wong et al., 2019).

In Japan, most psychiatric care is still provided in inpatient settings (WHO, 2020) despite the efforts to move on from inpatient care to community mental health care. Even though psychosocial interventions play an important role among patients with schizophrenia (McDonagh et al., 2022), there is a strong emphasis on pharmacological treatments rather than psychosocial treatments (OECD, 2015). In addition, little is known about the modifiable factors that psychosocial treatments should target. This study aimed to investigate the association between self‐stigma and self‐compassion in patients with schizophrenia from admission to the first follow‐up after discharge. The findings from this study will provide suggestions for adopting psychosocial interventions for nurses working at psychiatric hospitals in Japan.

## 2 METHODS

We included patients with schizophrenia who were hospitalized in an acute psychiatric ward in a private psychiatric hospital in Japan. The inclusion criteria were as follows: (1) diagnosis of schizophrenia (ICD‐10 code F20) and (2) aged ≥20 years. The exclusion criteria were patients with (1) intellectual disabilities, (2) dementia, or (3) any condition in which the doctor decided that the patient was not capable of participating in the study. We selected diagnosis of schizophrenia as the primary inclusion criterion based on the assumption that schizophrenia represents a spectrum of psychotic disorders and psychotic symptoms typically associated with self‐stigma. The recruitment was conducted between October 2020 and August 2021.

We obtained written informed consent from patients for participation in the semi‐structured interview and for responding to the questionnaire 1 month after the admission. Based on our clinical experience, we determined that 1 month from admission was sufficient to confirm the irreversible diagnosis of schizophrenia. The participants attended the interview and answered the self‐report questionnaires including measures of self‐stigma and self‐compassion at three time points: 1 month after admission (T1), discharge (T2), and first follow‐up after discharge at outpatient care (T3). Participants who completed the survey received a 500‐yen prepaid card during their first outpatient care visit after discharge.

Self‐stigma was measured using the Japanese version of the Internalized Stigma of Mental Illness (ISMI) scale. It contains the following five subscales: alienation, stereotype endorsement, perceived discrimination, social withdrawal, and stigma resistance. The total score ranges from 29 to 116. The higher the ISMI scores of the person, the stronger is the internalized stigma (Ritsher et al., 2003; Tanabe et al., 2016).

Self‐compassion was measured using the Japanese version of the Self‐Compassion Scale (SCS). The SCS comprises the following six subscales: self‐kindness (range 5–25), self‐judgment (5–25), common humanity (4–20), mindfulness (4–20), isolation (4–20), and over‐identification (4–20). Self‐judgment, isolation, and over‐identification were reverse‐scored. A higher total score indicates a higher level of self‐compassion (Arimitsu, 2014; Neff, 2003).

Psychiatric symptoms were measured using the Japanese version of the Positive and Negative Syndrome Scale (PANSS). It includes the following three subscales: positive symptoms (range 7–49), negative symptoms (7–49), and general psychopathology (16–112). The positive and negative symptoms were used for the analysis, with higher scores indicating more severe symptoms (Kay et al., 1987; Yamada et al., 1993). A semi‐structured interview was performed with each patient using PANSS by the first author, a psychiatric nurse who had completed the requisite training and was supervised by a psychiatrist during the assessment.

Sociodemographic variables included sex, age at current admission, and level of education. Clinical characteristics included number of previous psychiatric hospitalization and total length of stay in previous psychiatric hospitalizations. Patients' experience with coercive measures have been suggested to cause self‐stigma in people with schizophrenia (Bachtiar et al., 2020; Eka & Daulima, 2009; Lee et al., 2006). Thus, to describe the nature of patients' experiences during the hospitalization, we also collected data on experience with coercive measures including previous experience requiring mechanical restraints in psychiatric hospitalizations, previous experience of seclusion in psychiatric hospitalizations, and other coercive measures used during the index hospitalization.

We examined the change in positive and negative symptoms, as well as in subscales of self‐compassion, across time of assessment using linear mixed models, with patients as the random effect and time of assessment as the fixed effect. A linear mixed model was also used to examine the association between self‐stigma and self‐compassion. The first model included positive and negative symptoms as the covariates. In the second model, six subscales of self‐compassion were added to the first model. To assess the level of contribution of self‐compassion to the model for self‐stigma, the local effect size between model 1 and 2 was calculated using Cohen's *f* ^2^. Cohen's *f* ^2^ values of ≥0.02, ≥0.15. and ≥0.35 approximately represent small, medium, and large effect sizes, respectively (Selya et al., 2012).

All statistical analyses were performed using the statistical software IBM SPSS Statistics version 21 (IBM; Armonk, NY, USA). Statistical significance was set at *p* < .05.

This study was approved by the Ethics Committee of Tohoku University Graduate School of Medicine (approval number: 2022‐1‐844) and was conducted in accordance with the Helsinki Declaration of 1975 (as revised in 2013).

## 3 RESULTS

Twenty‐nine patients agreed to participate in this study. Of the 29 participants, 6 withdrew consent due to a change in diagnosis (*n* = 1), relocation for treatment of physical complications (*n* = 1), and/or worse mental health conditions (*n* = 5). A total of 23 patients participated in the T1 assessment. Of these, 10 (43.5%) were men, and 17 (73.9%) had a history of psychiatric hospitalization. For the index hospitalization, 18 participants (78.3%) were admitted involuntarily. During the index hospitalizations, 10 participants (43.4%) had experienced seclusion (nine participants, 39.1%) or mechanical restraint (1 participant, 4.3%). Further details are provided in Table 1. At T2, three participants did not participate in the survey due to unplanned discharge, resulting in a total of 20 participants completing the assessment. At T3, two participants came to the first follow‐up but were unable to complete the questionnaires due to worsening symptoms and two participants did not attend the clinic as they were assigned to a different provider for the first follow‐up after discharge. The remaining 19 participants completed the T3 assessment. The average period of T1–T2 was 50.3 days, and the average period of T2–T3 was 26 days. During the treatment period, all patients received psychiatric evaluations 1–3 times per week and pharmacotherapy by the doctor, and 17 out of 23 patients participated in occupational therapy. None of the patients had psychosocial intervention with a focus on self‐stigma or self‐compassion.

**TABLE 1:** Characteristics of the study participants.

Characteristics	Number (%)
Gender	
Man	10 (43.5)
Woman	13 (56.5)
Age at current admission	
≤39 years	5 (21.7)
40–49 years	5 (21.7)
50–59 years	6 (26.1)
≥60 years	7 (30.4)
Level of education	
Junior high school	5 (21.7)
High school	7 (30.4)
Vocational school	2 (8.7)
College graduated	7 (30.4)
No data	2 (8.7)
Number of previous psychiatric hospitalizations	
Never	5 (21.7)
Once	1 (4.3)
Twice	3 (13.0)
3–5 times	8 (34.8)
6–9 times	2 (8.7)
10 times or more	3 (13.0)
Total length of stay in previous psychiatric hospitalizations	
Never	5 (21.7)
4–7 months	4 (17.4)
8–12 months	3 (13.0)
≥13 months	5 (21.7)
Information was unavailable	6 (26.1)
Previous experience requiring mechanical restraints in psychiatric hospitalizations	
Yes	3 (13.0)
No	20 (87.0)
Previous experience of seclusion in psychiatric hospitalizations	
Yes	5 (21.7)
No	18 (78.3)
Coercive measures used during the index hospitalizations	
Seclusion	9 (39.1)
Mechanical restraint	1 (4.3)
Never	13 (56.5)

*Note*: The length of hospital stay for most of the participants in this study was about 90 days due to the medical fee system in Japan.

The mean and standardized deviations of self‐stigma, self‐compassion, and positive and negative symptoms at each assessment time point are shown in Table 2. Self‐stigma, as measured by the total ISMI score, did not change over time. Psychiatric symptoms, as measured by the PANSS score, showed a significant change. Positive symptoms significantly decreased from T1 to T2 (*p* = .008), and from T1 to T3 (*p* = .002). Negative symptoms also significantly decreased from T1 to T2 (*p* = .011), and from T1 to T3 (*p* = .004). One subscale of self‐compassion, “over‐identification,” as measured by the SCS‐J score, significantly decreased from T1 to T3 (*p* = .013).

**TABLE 2:** Change in self‐stigma, self‐compassion, and positive and negative symptoms at each assessment time.

	T1	T2			T3		
	Mean (SD)	Mean (SD)	Coefficient (95% CI)	*p*‐value	Mean (SD)	Coefficient (95% CI)	*p*‐value
Self‐stigma (range 29–116)	63.70 (12.13)	63.80 (11.90)	−0.603 (−5.504, 4.299)	.805	62.26 (19.12)	−2.100 (−7.090, 2.898)	.401
Self‐compassion							
Self‐kindness (range 5–25)	15.52 (4.66)	14.15 (4.79)	−0.817 (−2.523, 0.888)	.338	14.42 (4.75)	−0.613 (−2.340, 1.134)	.487
Self‐judgment (range 5–25)	14.83 (5.59)	15.85 (4.04)	1.195 (−0.387, 2.776)	.134	15.84 (4.94)	1.547 (−0.065, 3.159)	.069
Common Humanity (range 4–20)	12.78 (3.97)	12.30 (3.95)	−0.233 (−1.935, 1.470)	.784	12.21 (4.32)	−0.509 (−2.242, 1.224)	.556
Isolation (range 4–20)	12.48 (3.87)	13.10 (3.93)	0.585 (−0.699, 1.870)	.362	13.63 (3.44)	1.203 (−0.106, 2.512)	.071
Mindfulness (range 4–20)	11.91 (3.72)	11.50 (3.62)	−0.208 (−1.649, 1.233)	.772	11.95 (3.63)	0.206 (−1.251, 1.673)	.777
Over‐identification (range 4–20)	10.91 (4.17)	11.15 (4.53)	0.183 (−1.130, 1.497)	.779	12.32 (4.12)	1.723 (0.385, 3.061)	.013
Psychiatric symptoms							
Positive symptoms (range 7–49)	11.65 (2.57)	9.90 (2.69)	−1.541 (−2.661, −0.421)	.008	9.47 (2.59)	−1.883 (−3.023, −0.742)	.002
Negative symptoms (range 7–49)	11.57 (4.60)	9.15 (4.27)	−2.348 (−4.136, −0.560)	.011	8.26 (4.18)	−2.742 (−4.563, −0.921)	.004

*Note*: T1: at one month after admission, *N* = 23. T2: at discharge, *N* = 20. T3: at the first follow‐up after discharge in outpatient care, *N* = 19. A linear mixed model was used to estimate coefficients and *p*‐values, including patients as a random effect and the time of assessment as a fixed effect. Self‐stigma was measured using the Japanese version of the International Stigma of Mental Illnesses Scale. Self‐compassion was measured using the Japanese version of the Self‐Compassion Scale. Positive and negative symptoms were assessed using the Japanese version of the Positive and Negative Symptoms Scale.

Abbreviation: SD, standard deviation.

The results of the mixed linear model are shown in Table 3. The first model showed that patients with more positive symptoms reported worsening self‐stigma (*p* = .052). The second model showed a significant association between worsening self‐stigma and higher over‐identification (*p* = .001). The Cohen's *f* ^2^ was 0.46, suggesting a large effect size (≥0.35).

**TABLE 3:** Association between self‐stigma and self‐compassion at 1 month after admission, discharge, and first follow‐up.

	First model		Second model	
	Coefficient (95% CI)	*p*‐value	Coefficient (95% CI)	*p*‐value
Psychiatric symptoms				
Positive symptoms (range 7–49)	1.214 (−0.100, 2.437)	.052	0.733 (−0.273, 1.739)	.150
Negative symptoms (range 7–49)	−0.247 (−0.999, 0.504)	.512	0.242 (−0.393, 0.877)	.448
Self‐compassion				
Self‐kindness (range 5–25)	–	–	−0.207 (−1.034, 0.618)	.616
Self‐judgment (rage 5–25)	–	–	−0.526 (−1.578, 0.525)	.319
Common humanity (range 4–20)	–	–	−0.438 (−1.509, 0.634)	.416
Isolation (range 4–20)	–	–	0.664 (−0.590, 1.917)	.293
Mindfulness (range 4–20)	–	–	−0.140 (−1.466, 1.185)	.832
Over‐identification (range 4–20)	–	–	−2.230 (−3.435, −1.025)	.001

*Note*: A linear mixed model was used, including patients as a random effect and the time of assessment as a fixed effect. Self‐stigma was measured using the Japanese version of the Internalized Stigma of Mental Illness Scale, with scores ranging from 29 to 116. The first model included positive and negative symptoms as the covariates. In the second model, we added self‐compassion (self‐kindness, self‐judgment, common humanity, mindfulness, isolation, and over‐identification) to the first model. Positive and negative symptoms were assessed using the Japanese version of the Positive and Negative Symptoms Scale. Self‐compassion was measured by the Japanese version of the Self‐Compassion Scale.

## 4 DISCUSSION

In this study, patients with lower over‐identification were more likely to report lower self‐stigma. People with severe positive symptoms exhibited a trend toward worse self‐stigma, consistent with a previous study (Lysaker et al., 2007). However, the association was attenuated by self‐compassion, which showed a large contribution to the model for self‐stigma. Over‐identification with psychosis may reflect the patients focusing exclusively on their suffering and being fixated on repetitive thoughts about their illness, as suggested by Barnard and Curry (2011). Furthermore, over‐identification was significantly decreased from admission to outpatient care settings, while the remaining subscales of self‐compassion did not show significant changes over time. The discharge from inpatient care settings might contribute to the reduction in patients' repetitive thinking, which could have been induced by inpatient care settings and professionals' attention toward psychosis. Therefore, targeting over‐identification will aid in psychosocial interventions to enhance self‐compassion and prevent self‐stigma among patients with psychosis. Psychosocial interventions that aim to reduce self‐stigma include psychoeducation (Ivezić et al., 2017; Uchino et al., 2012) and narrative enhancement and cognitive therapy (Yanos et al., 2019). However, such interventions do not necessarily encompass nourishing self‐compassion. Our findings agree with the implications of the previous studies suggesting that targeting over‐identification in stigma‐reduction interventions may be beneficial. Such an adaptation should be further developed and examined for its effectiveness in reducing self‐stigma among people with psychosis. Over‐identification may reflect a perception of transitory events as definitive and permanent (Neff, 2023). Mindfulness would mitigate such perception via a balanced awareness of the discomfort of present‐moment experience (Shapiro et al., 2007). Literature suggests that over‐identification may be modifiable through a mindfulness‐based treatment among people bereaved by suicide (Scocco et al., 2018). Mindfulness‐based therapies may also be effective in reducing self‐stigma among patients with schizophrenia (Yılmaz & Kavak, 2020). Self‐compassion with a balanced, mindful approach helps people step outside of suppressing or exaggerating difficult thoughts and feelings. Therefore, mindfulness is the pillar on which self‐compassion rests. Since some mindfulness‐based approaches are effective in enhancing self‐esteem and diminishing self‐stigma among patients with schizophrenia (Dai et al., 2024), such interventions with a specific focus on over‐identification may reduce self‐stigma in this population.

The absence of change in self‐compassion regardless of reduced psychotic symptoms may reflect insufficient non‐pharmacological interventions during the index hospitalization. Nurses may have a role in fulfilling patient needs by providing mindfulness‐based interventions to address over‐identification in patients with psychosis through a trusted and reflective nurse–patient relationship. The relationship is the primary means of providing psychosocial interventions (Ruch, 2005) and is often the basis of mental health nursing. Mental health nurses in hospitals provide 24‐h nursing to inpatients, aiming to respond and reflect the patient's needs, which requires a holistic understanding of the patient in unique, complex, and dynamic situations (Ruch, 2005). As nurse‐led therapy for individuals with mental disorders has the potential to improve self‐esteem and other clinical outcomes (Kunikata et al., 2021), nurse‐led psychosocial interventions focusing on over‐identification and reducing self‐stigma may also be promising.

### 4.1 Strengths and limitations

The main strength of this study is the repeated measurement of primary independent variables and outcomes. However, this study had some limitations. We recruited participants from one institution. Thus, the generalizability of our findings should be interpreted with caution. We did not adjust demographic and clinical characteristics in the analyses of self‐stigma. However, such characteristics are not necessarily modifiable, and their association with self‐stigma was beyond the scope of this work. Moreover, these variables were stable across time, as captured in random intercepts for each patient. Additionally, the impact of coercive measures during the index hospitalization needs further clarification. Minimizing the use of coercive measures has been part of the priority agenda in Japanese mental health policies. Therefore, integrating self‐stigma into the outcome measures of an effectiveness trial for coercive measure minimization would provide further insights into the relevant associations. Our follow‐up assessment was also limited to the first outpatient visit after the discharge. Levels of self‐compassion and self‐stigma can change over time while living in the community, as patients with schizophrenia may face challenges that they would not have expected during hospitalization. The long‐term trajectory of self‐compassion and self‐stigma needs to be further clarified in a multicenter longitudinal study.

## 5 CONCLUSIONS

To our knowledge, this study is the first to investigate the longitudinal association between self‐stigma and self‐compassion in patients with schizophrenia from psychiatric hospitalization until the first outpatient follow‐up after discharge. Our results suggest that interventions with a specific focus on over‐identification can reduce self‐stigma in people with psychosis. Mental health nurses can lead the implementation of such interventions based on their holistic understanding of the patient and the established nurse–patient relationship. Nurse‐led intervention programs with a focus on over‐identification should be further developed and examined for their effectiveness.

## AUTHOR CONTRIBUTIONS

Keita Toshi contributed to the conceptualization, data curation, formal analysis, investigation, methodology, resources, software, visualization, and writing – original draft preparation of this study. Miharu Nakanishi contributed to the formal analysis, methodology, software, supervision, visualization, and writing – review and editing of this study. Mai Sakai contributed to the supervision, validation, and writing – review and editing of this study. Hatsumi Yoshii contributed to the conceptualization, funding acquisition, methodology, project administration, resources, supervision, validation, visualization, and writing – review and editing of this study. All authors read and approved the final manuscript.

## CONFLICT OF INTEREST STATEMENT

Keita Toshi worked as a nurse at the participating hospital. All other authors declare no competing interests.
